# Trigeminal Neuralgia With Concomitant Continuous Pain Due to Vertebrobasilar Dolichoectasia: A Case Report

**DOI:** 10.7759/cureus.49953

**Published:** 2023-12-05

**Authors:** Miki Takei, Keita Takizawa, Akiko Okada, Naoki otani, Noboru Noma

**Affiliations:** 1 Department of Oral Medicine, Nihon University School of Dentistry, Tokyo, JPN; 2 Department of Neurological Surgery, Nihon University School of Medicine, Tokyo, JPN

**Keywords:** vertebral basilar artery, microvascular decompression, vertebrobasilar dolichoectasia, hemifacial spasms, trigeminal neuralgia with concomitant continuous pain

## Abstract

This passage discusses a case of trigeminal neuralgia (TN) with continuous pain and hemifacial spasm caused by vertebrobasilar dolichoectasia, a rare condition. The patient experienced ongoing orofacial pain, which initially led to dental treatments. After unsuccessful medication (carbamazepine), the patient underwent microvascular decompression to alleviate nerve compression by the elongated vertebral artery. This report highlights the challenge of treating such cases due to the unique nature of neurovascular compression. Additionally, it introduces the concept of TN with concomitant continuous pain and emphasizes the need for comprehensive diagnosis, as vertebrobasilar artery elongation is associated with various symptoms, including TN and hemifacial spasms.

## Introduction

Nerve root atrophy and/or displacement due to neurovascular compression are independently associated with the signs and symptoms of trigeminal neuralgia (TN). When these anatomical changes are present, the condition is diagnosed as classical TN (CTN) [1]. In 2020, the International Classification of Orofacial Pain (ICOP) classified CTN as "CTN, purely paroxysmal” and “CTN with concomitant continuous pain” [2]. CTN with concomitant continuous pain manifests as ongoing background pain between paroxysmal attacks, which is commonly described as aching, throbbing, or burning.

Craniocerebral nerve compression caused by an elongated vertebrobasilar system has been documented as a contributing factor in TN and hemifacial spasm [3]. Some researchers reported CTN and hemifacial spasms attributed to vascular compression by the vertebrobasilar system but not CTN with concomitant continuous pain caused by the vertebrobasilar system [4,5]. In this report, we present a case of a CTN with concomitant continuous pain and hemifacial spasm due to an elongated vertebrobasilar system.

## Case presentation

A 59-year-old man presented with a chief complaint of dull, continuous, aching pain of five months' duration in the left maxillary molar areas and one month of involuntary spasms of the left side of the face involving perioral and periocular muscles. The patient had consulted a dentist for treatment of aching pain in the same region. Although he had undergone tooth extraction (from the left maxillary second premolar to the left maxillary second molar) based on the diagnosis of periodontal disease, the pain continued. Two months earlier, the patient consulted an oral and maxillofacial surgeon who diagnosed upper left wisdom tooth pericoronitis, and this tooth was extracted. Approximately one month after undergoing wisdom tooth extraction, the patient started experiencing continuous intense pain, along with shooting-like pain lasting for one minute, from the left infraorbital area to the cheek when eating, talking, and brushing teeth. It was accompanied by spasms of the left side of the face, lasting for a few seconds, once a day. The patient also complained of twitching over the left eyelid and forehead three months prior. For a month, the frequency is a couple of times a day. The patient was referred to an ear, nose, and throat specialist and prescribed antibiotics and pregabalin (150 mg/day), but since the pain did not improve, the patient was subsequently referred to our clinic.

The patient had a significant history of hypertension over the past five years. The patient was a heavy smoker in the past, experienced a cerebral hemorrhage five years ago, and underwent surgery.

**Figure 1:**
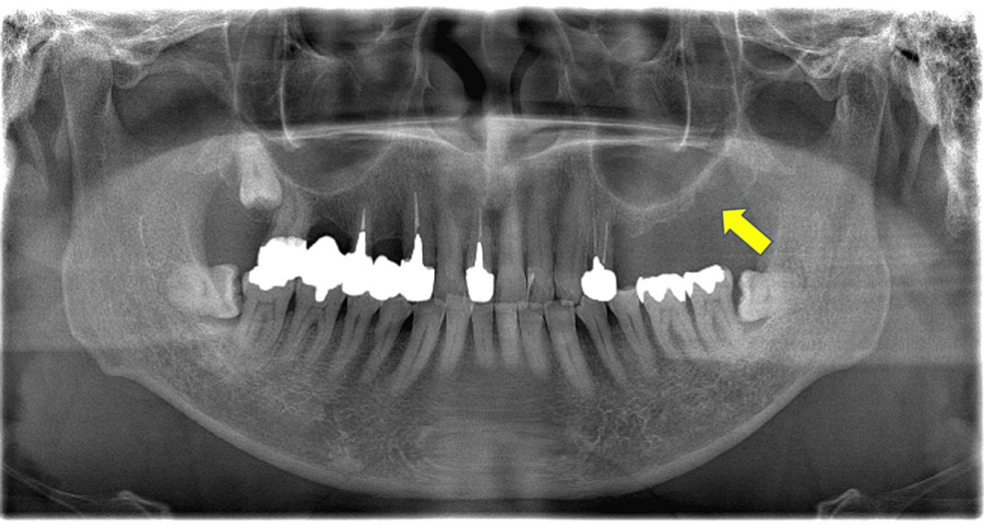
Panoramic radiographs A defect in the left upper molar region is observed, and no abnormalities are noted within the maxillary sinus.

On neurologic examination, the temporomandibular joint, masticatory muscle, and intraoral findings were within normal limits. A light touch with a cotton swab to the left cheek area provoked sharp, lancinating pain that persisted for a few seconds, and when the skin was lifted upward, it elicited pain and facial twitching. Panoramic radiography revealed a loss of teeth from the left maxillary second premolar to the left maxillary second molar and resorption of the maxillary tuberosity (Figure 1).

**Figure 2:**
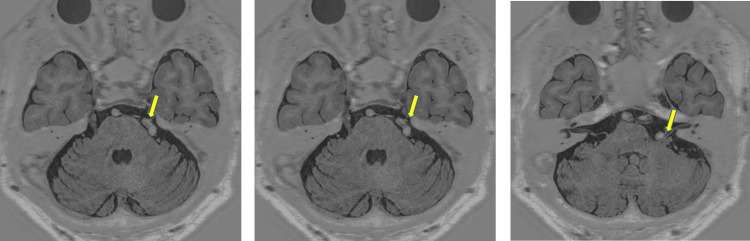
MRI of the head The left vertebral artery is dilated and tortuous, compressing the left trigeminal nerve strongly on the peripheral side after its branching from the brainstem in an upward direction.

**Figure 3:**
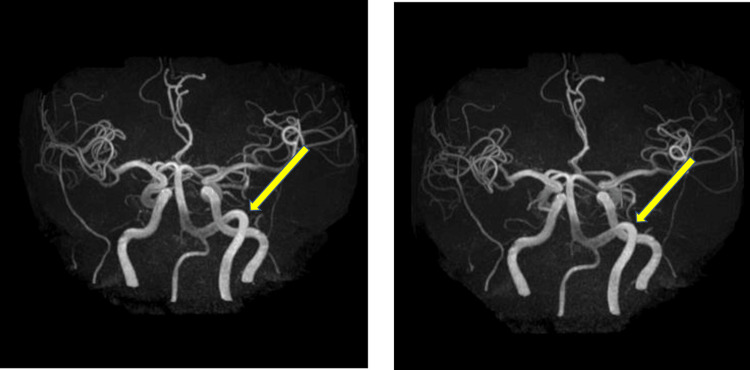
MRA of the head Left vertebral angiogram demonstrating the tortuous course of the vertebral artery

Magnetic resonance imaging (MRI) and magnetic resonance angiography (MRA) revealed a significantly dilated and tortuous vertebrobasilar artery that was contacting the left trigeminal root entry zone and facial nerve (Figures 2-3).

He was diagnosed as having TN and started on carbamazepine 100 mg twice daily, with which the paroxysmal pain resolved. However, two weeks later, pain control became challenging, and the dosage was increased to 200 mg twice a day, but the medications were not effective anymore, leading to a referral to the neurosurgery department.

**Figure 4:**
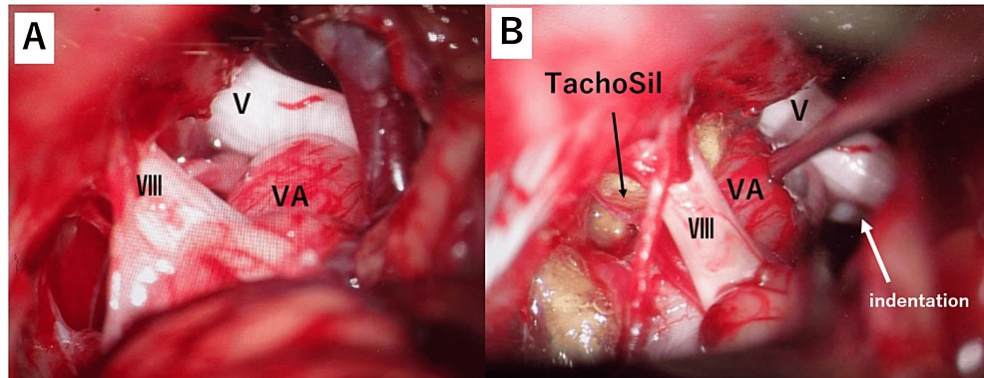
Intra-operative image (around the REZ of the trigeminal nerve) (A) Intra-operative image demonstrating the medulla with the VA, which is compressing V (the trigeminal root entry zone). (B) Intra-operative image showing the area around the REZ of the trigeminal nerve after the repositioning of the VA. Following the VA away from the medulla, indentations on the trigeminal nerve root were observed. V: trigeminal nerve, Ⅶ.Ⅷ: facial nerve, REZ: root entry zone, TachoSil: TachoSil tissue sealing sheet, VA: vertebral artery

The patient was considered for a surgical procedure to alleviate the neurovascular compression. An informed consent was obtained, and the procedure was performed. The patient was informed about the surgery step by step, its benefits, potential side effects, and complications that could arise during and after the surgery. This procedure was performed using a retrosigmoid approach, bordered superiorly by the transverse sinus and laterally by the sigmoid sinus. The cerebellar hemisphere was retracted, and the arachnoid membrane was dissected. The dolichoectatic vertebral artery compresses the trigeminal root entry zone along the lateral side of the V and VII/VIII complex (Figure 4).

**Figure 5:**
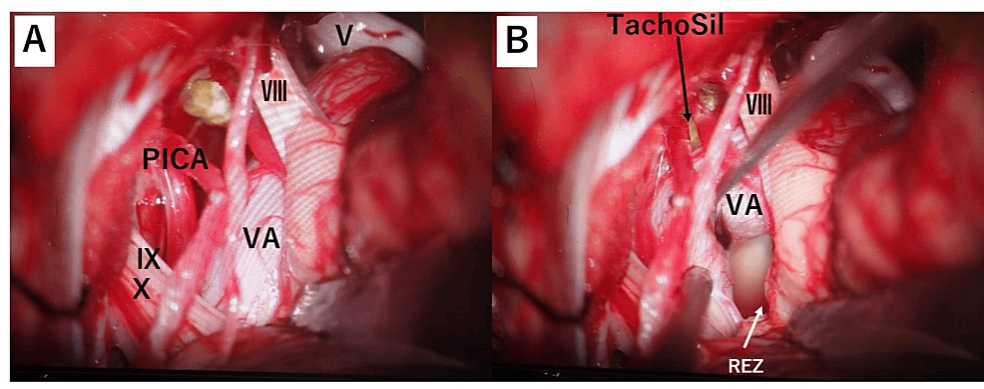
Intra-operative image (around the REZ of the facial nerve) (A) Intra-operative image demonstrating the medulla with the VA, which is compressed Ⅶ.Ⅷ. (B) Intra-operative image showing the area around the REZ of the facial nerve after the repositioning of the VA. Following the VA away from the medulla, indentations on the trigeminal nerve root were observed. V: trigeminal nerve, Ⅶ.Ⅷ: facial nerve, Ⅸ: glossopharyngeal nerve, X: vagus nerve, PICA: the posterior inferior cerebellar artery, REZ: root entry zone, TachoSil: TachoSil tissue sealing sheet, VA: vertebral artery

The dolichoectatic vertebral artery was so hard and immobile that it was very difficult to decompress the nerve in the usual manner. Therefore, the approach was made from the lower brainstem level of the medulla to the vertebral artery trunk, and by inserting and suspending a Teflon felt, the vertebral artery was gradually repositioned as a whole. The decompression procedures for the trigeminal nerve and facial nerve were completed (Figure 5).

Immediately after the surgery, the paroxysmal pain and hemifacial spasms disappeared. There were no postoperative complications, such as hearing loss or facial nerve paralysis, and the patient was able to walk independently within two weeks.

## Discussion

We report a case of vertebrobasilar dolichoectasia, a very rare cause of TN and hemifacial spasm. Vertebrobasilar dolichoectasia involves abnormal elongation and expansion of arteries that typically affect the posterior intracranial circulation. Although the etiology of this condition remains unclear, it has been suggested to be associated with other disorders such as ischemic attacks, ischemic strokes, and subarachnoid hemorrhages [6].

This is often observed in the elderly population, especially in those with concomitant hypertension, and it is associated with significant arterial atherosclerotic changes in the arterial intima. It is more commonly seen in males, and when it affects one side, it is often on the left side and may also be accompanied by a hemifacial spasm on the same side [7]. Vertebrobasilar dolichoectasia can be asymptomatic in some cases. In cases with symptoms, the clinical manifestations can vary widely [6]. The most common clinical symptom is an ischemic stroke. Other symptoms include those related to cranial nerve compression, brainstem compression, bleeding, and obstructive hydrocephalus [8]. In this case, the patient is a 59-year-old male with a history of hypertension and cerebral hemorrhage. He developed TN and hemifacial spasms related to neurovascular compression of cranial nerves (trigeminal and facial nerves) at the root entry zone.

Ten cases, including ours, have been reported for TN and hemifacial spasms due to vertebrobasilar dolichoectasia over the decade (2013-2023) (Table 1) [9-15].

**Table 1:** Ten cases of TN and hemifacial spasms due to vertebrobasilar dolichoectasia over the decade (2013-2023) m: male, f: female, NA: not applicable, MVD: microvascular decompression, VA: vertebral artery, TN: trigeminal neuralgia, HFS: hemifacial spasm, GPN: glossopharyngeal neuralgia

Previous report	Age	Sex f/m	Duration	Treatment	Outcome
2013 Lakhan [9]	64	m	2 years	Gamma Knife radiosurgery for pain, botulinum neurotoxin injection for spasms. Conservative medical treatments like carbamazepine, oxcarbazepine, and levetiracetam were unsuccessful	The patient opted for Gamma Knife radiosurgery, providing months of pain relief, and was later offered botulinum neurotoxin injections for spasms
2016 Revuelta-Gutiérrez et al. [10]	Case 1: 64, case 2: 75	Case 1: m, case 2: f	Case 1: 2-4 years, case 2: 2 years	Case 1: MVD. Case 2: MVD techniques were used: a microasterional approach and Teflon in 2 cases	After surgery, both cases experienced complete resolution of symptoms, with immediate cessation of paroxysmal pain and involuntary movements
2018 Han et al. [11]	66	f	3 years	Pharmacotherapy, carbamazepine, vitamin B1, methylcobalamin	Symptoms improved after 7 days of treatment, but 5 months later, a right HFS developed. No further treatment; lost follow-up after 6 months
2019 Rodriguez et al. [12]	79	m	4 months	NA	An MRI with angiography showed a vertebrobasilar dolichoectasia causing compression of the left V, VII, and VIII cranial nerves
2020 Perez-Roman et al. [13]	66	f	NA	The MVD method was used, and the clip-sling technique was used to achieve MVD	The patient had immediate complete relief from TN, HFS, and GPN postoperatively
2022 Liu et al. [14]	Case 1: 67, case 2: 79	Case 1: m, case 2: m	Case 1: 2.5 years, case 2: 6 months	MVD, a novel technique was described, involving the anterolateral mobilization of the vertebrobasilar system toward the occipital condyle using a sling for brainstem decompression	Both patients had immediate radiographic improvement in the degree of brainstem compression and significant improvement in presenting symptoms at 2 months follow-up
2022 Alotaibi et al. [15]	77	f	2 years	Pharmacotherapy, vertebrobasilar artery dilatation improved by medical therapy with carbamazepine	Initiate medical treatment first; it may be effective, avoiding surgery, especially in comorbid patients ineligible for surgical intervention
2023 (our case) Takei et al.	59	m	5 months	MVD: approaching the VA trunk from the lower brainstem nerve level and using techniques such as the insertion and suspension of Teflon felt (TachoSil) allows for gradual displacement of the VA	Immediately after the surgery, the paroxysmal pain and HFS disappeared. There were no postoperative complications, such as hearing loss or facial nerve paralysis

A previously reported review of TN and hemifacial spasms due to vertebrobasilar dolichoectasia revealed an age range of 59 to 79 years, with six males and four females. Regarding treatment, there were six cases of microvascular decompression, two cases of medical therapy, one case of Gamma Knife radiosurgery (and botulinum neurotoxin injection), and one case with no treatment documented. Previously, various surgical techniques have been outlined for the management of vascular compression. These include an anteromedial directed muslin sling with clip-assisted tethering to the clival dura [16], a transcondylar fossa approach to laterally sling the vertebral artery using a sling stitched to the dura of the jugular tubercle [17], and a "strip-clip" technique developed by Raabe et al., employing an equine collagen strip and mini-aneurysm clips to decompress the vertebral artery from the brainstem. Additionally, the use of a Gore-Tex sling on the petrous ridge has been reported to alleviate hemifacial spasms [18]. Furthermore, procedures such as vessel sectioning and bypass have been detailed for the decompression of cranial nerves [19]. Liu et al. reported a novel surgical technique involving anterolateral mobilization of the vertebral artery. This method provides the flexibility to either stitch a suture to the dura of the occipital condyle or use a cranial fixation screw directly on the condyle, serving as a secure anchor [14].

In neurovascular decompression procedures such as microvascular decompression, it is crucial to adequately move the responsible compressing blood vessels to relieve pressure on the nerves. However, in cases of vertebrobasilar dolichoectasia, the vascular system is often tortuous and rigid throughout, making it challenging to move the compressing responsible vessels significantly by only addressing the higher portions of neurovascular compression. Therefore, as was done in this case, approaching the vertebral artery trunk from the lower brainstem nerve level and using techniques such as the insertion and suspension of Teflon felt (TachoSil) allows for gradual displacement of the vertebral artery. This, in turn, makes it possible to reposition the compressing responsible vessels like the vertebral artery and the posterior inferior cerebellar artery at higher levels, enabling the decompression of the trigeminal and facial nerves. In this surgery, there were no complications; however, generally, there is a potential risk of facial and auditory nerve damage. Therefore, in cases of TN and hemifacial spasms associated with the vertebrobasilar artery, a broader surgical approach is necessary to ensure a comprehensive view of the neurovascular anatomy, often requiring a wider operative field than usual.

The term "pre-trigeminal neuralgia (PTN)" was coined by Fromm et al. to describe persistent facial pain that precedes paroxysmal pain in TN [20]. The pain associated with PTN is felt in the teeth, alveolar bone, and/or sinus regions. PTN is often described as a dull ache, sharp, shooting, or lancinating pain, although reports vary in their descriptions. This pain can be continuous or episodic, lasting for minutes to several hours, and it may persist for days to years before typical TN symptoms appear. In the ICOP classification, the terms "typical TN symptom with background pain including PTN," "atypical TN," and "TN type 2" have been replaced with "CTN with concomitant continuous pain" [1]. This case represents the first report of "CTN with concomitant continuous pain" caused by vertebrobasilar dolichoectasia. "CTN with concomitant continuous pain" closely mimics odontogenic pain, leading the patient to undergo tooth extractions before seeking treatment with a neurosurgeon. Primary care physicians and orofacial pain specialists should consider a comprehensive diagnosis, as vertebrobasilar artery elongation is often associated with hypertension and can manifest symptoms such as ischemic strokes, TN, and hemifacial spasms.

## Conclusions

TN due to vertebrobasilar dolichoectasia often presents with intense and intractable pain that can be difficult to control with medication (such as carbamazepine). The intense pain in this case may be due to the vertebral artery having a thicker vascular structure (resulting in greater pressure due to the compression area and volume) compared to the superior cerebellar artery and anterior inferior cerebellar artery. This strong compression is presumed to be the cause of the severe pain based on surgical observations. TN with vertebrobasilar dolichoectasia is a relatively rare condition and may be accompanied by hemifacial spasms. The conditions often mimic odontogenic pain. An accurate diagnosis is the key to preventing unnecessary dental treatments.
